# Pre-sleep milk-derived proteins do not impair sleep or recovery in elite female athletes

**DOI:** 10.1080/15502783.2026.2746899

**Published:** 2026-10-20

**Authors:** Kieran G. P. Paterson, Timothy D. Griest, Elisa Angeles, Sadio P. Fenner, Hannah K. Eberhardt, Connor R. McKay, Kendall L. W. Helms, Andelino Calderon, Matthew D. Vukovich, Michael J. Ormsbee

**Affiliations:** a Department of Health, Nutrition, and Food Sciences, Florida State University, Tallahassee, FL, USA; b Institute of Sports Sciences & Medicine, Florida State University, Tallahassee, FL, USA; c College of Education and Human Sciences, South Dakota State University, Brookings, SD, USA; d School of Health Sciences, Discipline of Biokinetics, Exercise and Leisure Sciences, University of KwaZulu-Natal, Durban, South Africa

**Keywords:** Pre-sleep feeding, macronutrient timing, female athletes, sleep quality, protein supplementation, heart rate variability

## Abstract

**Background:**

Sleep is essential for athlete recovery and performance, yet athletes frequently experience restricted or poor-quality sleep. Pre-sleep feeding (PSF) has emerged as a strategy to support recovery, but randomized, double-blinded, crossover trials comparing PSF macronutrient options on objective and subjective sleep outcomes in elite female athletes remain limited.

**Methods:**

Twenty-five National Collegiate Athletic Association (NCAA) Division I female athletes (18–25 years old) were recruited; 21 completed the trial. In a randomized, double-blinded, placebo controlled, crossover design conducted over 4–8 weeks, participants consumed four treatments for three consecutive nights each: 40 g casein protein (CAS; 160 kcal), 40 g *α*-lactalbumin protein (ALA; 160 kcal), 40 g carbohydrate (CHO; 160 kcal), and 40g of a placebo (PLA; 5 kcal), with a minimum three-day washout between conditions. Supplements were consumed ~2 h after the final meal and ~30 min before bedtime as the last caloric intake of the day. Objective outcomes were obtained from the WHOOP 4.0 device (total sleep time (TST), sleep onset latency (SOL), sleep efficiency (SE), resting heart rate (RHR), and heart rate variability (HRV)). Subjective outcomes were assessed each morning via questionnaires (subjective sleep quality (SSQ), subjective sleep onset latency (SSOL), and subjective recovery (SREC)). Nocturnal interstitial glucose was assessed via blinded continuous glucose monitoring (CGM) in a subset (*n* = 6). Sleep and recovery variables were analyzed using one-way repeated-measures analysis of variance (ANOVA) to evaluate treatment effects across the four supplementation conditions. For CGM data, a two-way repeated-measures ANOVA was used to evaluate main effects of treatment and time, as well as the treatment × time interaction. Tukey’s post hoc testing was used with significance set at *p* < 0.05.

**Results:**

No significant treatment effects were observed for TST, SOL, SE, RHR, or HRV (*p* > 0.05). TST ranged from 7.47–7.62 h, SE from 91.5–92.0%, RHR from 53.5–54.1 bpm, and HRV from 98.2–103.9 ms across conditions. No significant treatment effects were detected for SSQ, SSOL, or SREC (*p* > 0.05); SSQ ranged from 69–75%, SSOL from 16–20 min, and SREC from 61–66%. In the CGM sub-analysis, a main effect of time was observed for nocturnal glucose (*p* = 0.008), but no differences among treatments (*p* = 0.187) or a time × treatment interaction (*p* = 0.550) were detected.

**Conclusions:**

In NCAA Division I female athletes, three consecutive nights of pre-sleep ingestion of CAS, ALA, or CHO did not impair objective or subjective measures of sleep or recovery compared with PLA. These findings demonstrate that pre-sleep feeding, regardless of macronutrient type, can be incorporated without negatively affecting sleep quality or recovery metrics.

**Trial Registration:**

ClinicalTrials.gov (Identifier: STUDY00004409).

## Graphical abstract

**Figure:**
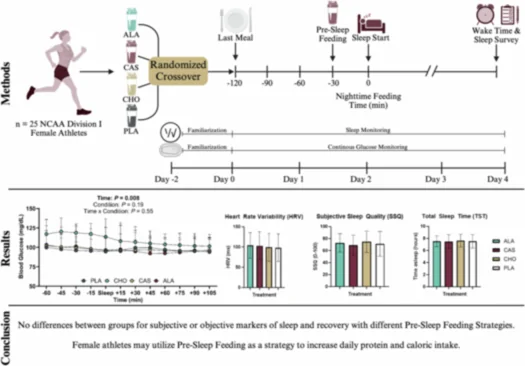

## 1. Introduction

Sleep is essential for athlete recovery and performance, yet athletes frequently experience restricted or poor-quality sleep due to late training, travel, and lifestyle demands [1–4]. Consensus recommendations further highlight that athletes may require 8–10 hours of sleep per night, exceeding the typical 7–9 hours recommended for the general adult population, although many still fall short of these needs [5,6].

A growing body of work links diet composition and timing with sleep physiology. For example, high-glycemic carbohydrate meals consumed several hours before bedtime can shorten sleep-onset latency, though findings remain inconsistent across studies [7–9]. In team sport athletes, habitual nutrient intake and evening meal patterns have been positively associated with greater sleep efficiency and duration [10,11]. In addition, athletes may be at a disadvantage if they do not eat following an exhaustive evening match or game, particularly when they must compete again within eight hours [12]. These observations suggest that nutrition may influence sleep, but controlled trials directly testing this relationship in athletes remain limited.

Pre-sleep feeding (PSF), defined as intentional ingestion of nutrients approximately two hours after dinner and ~30 minutes before bedtime, is one strategy used to support recovery. While the public’s perception of late-night eating as detrimental, especially for weight management and sleep quality, remains, evidence suggests that context is key to interpreting the data [13–15]. When protein-centric, PSF may provide a final opportunity to stimulate muscle protein synthesis (MPS) and support overnight recovery without impairing sleep [16–18]. Our group and others have shown that PSF of protein can influence resting energy expenditure, hunger, and metabolic markers without compromising exercise performance or recovery in active populations [19–21]. Importantly, a recent field study in NCAA Division I female soccer players reported that higher pre-sleep energy and protein intake did not influence recovery metrics such as heart rate variability and resting heart rate as measured by a wearable device, or measures of subjective recovery [22].

Within protein-based PSF, *α*-lactalbumin (ALA) has drawn interest due to its high tryptophan content, which may enhance serotonin and melatonin production and reduce sleep-onset latency [23]. Indeed, two trials have shown improvements in sleep architecture and next-morning function in female rugby athletes when ALA was consumed prior to sleep [24,25], whereas others have reported no benefit of ALA compared with energy-matched comparators in athletes with sleep difficulties [26]. However, the heterogeneity of study designs and populations studied limits the ability to draw strong conclusions in elite sport [27–30].

Despite these developments, there remains a lack of randomized, double-blinded, crossover trials directly comparing distinct PSF macronutrient options on both objective and subjective sleep outcomes in elite female athletes. This is important because female athletes may have unique physiological and hormonal influences on sleep, including menstrual-cycle-related autonomic differences [31,32].

Therefore, the purpose of this study was to examine the effects of three consecutive nights of pre-sleep ingestion of casein protein (CAS), *α*-lactalbumin protein (ALA), carbohydrate (CHO), and a placebo (PLA) on sleep and recovery outcomes in NCAA Division I female athletes. We hypothesized that ALA would elicit superior sleep and recovery outcomes compared with CAS and CHO, and that all nutrient interventions would outperform PLA.

## 2. Methods

### 2.1. Participants

Twenty-five female collegiate athletes (age: 18–25 years) were recruited from National Collegiate Athletic Association (NCAA) Division I teams at Florida State University. All participants wore a WHOOP 4.0 device (WHOOP Boston, MA, USA) continuously on their wrist for at least three weeks prior to enrollment as part of team monitoring programs. Exclusion criteria included diagnosed sleep disorders, disordered eating, or known allergies to any study supplement. Participants represented multiple sports, including women’s soccer (*n* = 12), softball (*n* = 4), and tennis (*n* = 5), reflecting varied training demands typical of NCAA Division I athletes. Participants were considered elite athletes according to the classification framework of McKay et al. [33], which defines ‘elite/international level’ as Tier 4 within a six-tier model of athlete caliber [33].

Menstrual cycle regularity and contraceptive use were self-reported using a study-specific questionnaire. Participants classified their cycles as regular (occurring every 21–35 days or ≥5 periods in the last 6 months) or irregular (cycles <21 days, >35 days, or fewer than 6 periods per year). These definitions align with established criteria for eumenorrheic and irregular cycles in female athletes [34].

Written informed consent was obtained from all participants before participation. The study was approved by the Florida State University Institutional Review Board (STUDY00000058) and registered at ClinicalTrials.gov (Identifier: STUDY00004409).

### 2.2. Study design and procedures

A randomized, double-blinded, placebo-controlled, crossover design was used over a 4–8-week period. The variable total duration reflected differences in scheduling between athletic teams and washout requirements. Each participant consumed four treatments in a random order: 40 g of casein protein (CAS; 160 kcal), 40 g of *α*-lactalbumin protein (ALA; 160 kcal), 40 g of carbohydrate (CHO; 160 kcal), and a placebo (PLA; 5 kcal). The PLA contained flavoring agents, non-nutritive sweeteners (sucralose, stevia Reb A), organic acids (citric and malic acid), and natural colorants (spirulina and red beet powder). The treatment order was determined using a computer-generated random sequence. All supplements were pre-packaged, blinded, and coded by Actus Nutrition (Eden Prairie, MN, USA) to ensure similar appearance, taste, and texture. Each condition consisted of three consecutive nights of supplementation followed by a minimum three-day washout period.

Following consent, participants completed baseline screening questionnaires regarding medical history, menstrual status, food history, and the Athlete-Specific Sleep Questionnaire (ASSQ). Participants arrived at the Institute of Sports Sciences and Medicine (ISSM) at least three hours fasted, abstained from caffeine, and avoided high-intensity exercise for 12 hours before their initial visit. At this visit, participants underwent dual-energy X-ray absorptiometry (DXA; Hologic Inc., Bedford, MA, USA) to assess body composition and bone mineral density.

### 2.3. Testing procedures

To assess for nocturnal interstitial glucose, Abbott Freestyle Libre Pro continuous glucose monitors (CGM) (Abbott Diabetes Care, Alameda, CA, USA) were applied to the posterior, nondominant upper arm at the start of each treatment period, approximately two days before treatment to allow sensor stabilization and resolution of local tissue irritation from insertion, thereby improving accuracy during data collection, and removed following each treatment. Sensor adhesion was monitored throughout each treatment period, although the athletic demands of training and competition occasionally resulted in premature detachment or loss of devices. Participants then received their assigned treatment with written and verbal instructions for preparation and timing. Supplements were provided as pre-portioned powdered doses in sealed, opaque bottles, each containing a fill line corresponding to 237 mL of water when mixed. Participants consumed the assigned supplement under free-living conditions.

Each treatment night, participants recorded all daytime food and beverage intake using a photographic food and beverage log that was sent electronically at 21:00. Logs excluded the intervention supplement, as participants were blinded to their assigned condition. Participants consumed their assigned supplement approximately two hours after their final meal and 30 minutes before bedtime as the last caloric intake of the day, with no additional foods or beverages co-ingested. Participants slept in their usual environments while continuously wearing the WHOOP 4.0 device. Compliance with supplementation and protocol timing was verified via supplement logs and CGM data. The procedure was repeated for three consecutive nights during each treatment period.

### 2.4. Sleep and recovery assessments

Upon waking after each treatment night, participants completed a subjective sleep questionnaire to assess perceived sleep quality and quantity. This was delivered electronically at 04:00 each morning to ensure the link was ready upon waking up. Participants were instructed to complete the SSQ within 30–60 minutes of waking.

On the morning after the third treatment night (Day 4), research staff removed the CGM devices and downloaded the data. Participants completed a brief questionnaire assessing menstrual-cycle day and perceived supplement identity to verify treatment blinding. Testing sessions were not standardized by menstrual cycle phase to ensure timely completion of all treatments within the competitive season.

A minimum three-day washout period followed each condition before the next treatment phase. This process was repeated until all four interventions (CAS, ALA, CHO, PLA) were completed in randomized order.

### 2.5. Outcome measures

Objective sleep and recovery outcomes were obtained from the WHOOP 4.0 device and included total sleep time (TST), sleep onset latency (SOL), sleep efficiency (SE), resting heart rate (RHR), and heart rate variability (HRV). Subjective outcomes were collected each morning through the electronic subjective sleep questionnaire and included subjective sleep quality (SSQ), subjective sleep onset latency (SSOL), and subjective recovery (SREC).

Dietary intake on intervention days was recorded using the photographic food and beverage log and analyzed in the Automated Self-Administered 24-Hour Dietary Assessment Tool (ASA24; National Cancer Institute, Bethesda, MD, USA) by trained research staff.

At the end of each treatment period, participants reported their current menstrual-cycle day and the supplement they believed they had consumed that week to assess treatment blinding accuracy.

### 2.6. Statistical analysis

Sleep and recovery variables were analyzed using one-way repeated-measures analysis of variance (ANOVA) to evaluate treatment effects across the four conditions. Continuous glucose monitoring outcomes were analyzed using two-way repeated-measures ANOVA to evaluate main effects of treatment and time, as well as the treatment × time interaction. Where appropriate, Tukey’s post hoc tests were performed. All analyzes were conducted using R (version 4.3.1; R Foundation for Statistical Computing, Vienna, Austria). Results are presented as mean ± standard deviation (SD), along with Cohen’s d effect sizes and 95% confidence intervals. Statistical significance was set at *p* < 0.05. An a priori power analysis (G*Power 3.1), based on previously reported effect sizes for sleep onset latency, indicated that 18 participants were required to achieve 80% power at *α* = 0.05. To account for potential attrition, 25 athletes were recruited.

## 3. Results

Twenty-one female athletes completed the trial. Participant characteristics are presented in Table 1. Two participants self-reported using sleep aids on an average of 5–7 nights per week. Eight participants (38%) were using hormonal contraception (HC), and thirteen were not (62%). Of those using HC, three reported oral contraceptive use, three used an intrauterine device (IUD), and two used a subdermal implant. Self-reported menstrual cycle regularity indicated that eight participants had irregular cycles and thirteen had regular cycles, according to the definitions above.

**Table 1.** Participant characteristics at baseline (*n* = 21 NCAA Division I female athletes).

Variable	Mean ± SD
Age (years)	21 ± 2
Height (m)	1.7 ± 0.1
Body mass (kg)	66.8 ± 11.9
Body fat (%)	27.3 ± 4.6
Body Mass Index (kg/m^2^)	23.3 ± 3.0
Fat Free Mass Index (kg/m^2^)	16.9 ± 1.7
Sport distribution (*n*)	Soccer = 12; Softball = 4; Tennis = 5

### 3.1. Sleep and recovery metrics

Objective sleep and recovery measures assessed via the WHOOP 4.0 device are shown in Table 2. No significant treatment effects were observed for total sleep time (TST), sleep onset latency (SOL), sleep efficiency (SE), resting heart rate (RHR), or heart rate variability (HRV) *(p* > 0.05). TST was not different across treatments, with values ranging from 7.47–7.62 h. SE remained consistently high (91.5–92.0%), while RHR (53.5–54.1 bpm) and HRV (98.2–103.9 ms) also showed no significant differences among conditions. RHR and HRV were averaged from nightly WHOOP-derived sleep values, reflecting overnight autonomic recovery rather than morning spot measures.

**Table 2.** Objective sleep and recovery outcomes (WHOOP 4.0) across treatments.

Variable	ALA	CAS	CHO	PLA
Total sleep time (hours)	7.53 ± 0.89	7.47 ± 1.14	7.62 ± 1.06	7.52 ± 1.11
Sleep onset latency (min)	2.27 ± 3.76	2.52 ± 4.19	1.96 ± 3.21	3.29 ± 4.65
Sleep efficiency (%)	92.02 ± 2.98	91.80 ± 3.16	91.50 ± 4.72	91.56 ± 3.21
Resting heart rate (bpm)	53.54 ± 6.19	53.71 ± 5.42	54.05 ± 6.66	53.71 ± 6.55
Heart rate variability (ms)	103.91 ± 31.48	103.05 ± 34.60	99.14 ± 34.08	98.24 ± 34.01

Note: Data are presented as mean ± standard deviation.

Abbreviations: ⍺-Lactalbumin (ALA), Casein (CAS), Carbohydrate (CHO), Placebo (PLA), Beats Per Minute (BPM), Milliseconds (MS).

Subjective sleep outcomes, including subjective sleep quality (SSQ), subjective sleep onset latency (SSOL), and subjective recovery (SREC), are presented in Table 3. No significant treatment effects were detected for any subjective measure (*p* > 0.05). SSQ scores did not differ among treatments, ranging from 69% to 75%. SSOL values were similar across conditions from 16 to 20 min, and SREC scores ranged from 61% to 66%, with no significant differences between treatments.

**Table 3.** Subjective sleep and recovery outcomes across treatments.

Variable	ALA	CAS	CHO	PLA
Subjective sleep quality (%)	72.92 ± 15.39	69.12 ± 16.66	74.88 ± 17.81	71.31 ± 20.60
Subjective sleep onset latency (min)	20.21 ± 16.83	18.08 ± 18.71	16.45 ± 12.28	16.62 ± 14.08
Subjective recovery (%)	64.73 ± 17.06	60.93 ± 19.76	65.64 ± 19.22	65.93 ± 20.40

Note: Data are presented as mean ± standard deviation.

Abbreviations: ⍺-Lactalbumin (ALA), Casein (CAS), Carbohydrate (CHO), Placebo (PLA).

### 3.2. Continuous glucose monitoring

Complete continuous glucose monitoring (CGM) data were available for six of the twenty-one participants across all four treatments, permitting a sub-analysis of nocturnal glucose responses. The reduced CGM sample was primarily due to sensor loss during training and competition activities, as frequent clothing changes, contact exposures, and diving movements in certain sport positions led to premature detachment or device removal. As a result, some individual treatment periods lacked valid recordings, preventing full four-condition comparisons. CGM data were used to verify supplement compliance and to evaluate overnight glucose trends. A significant main effect of time was observed for nocturnal glucose (*p* = 0.008), reflecting the expected physiological rise in glycemia before sleep, followed by a gradual decline across the night (Figure 1). However, no significant differences were detected among treatments (*p* = 0.187), nor was there a significant time × treatment interaction (*p* = 0.550), indicating that pre-sleep macronutrient type did not alter overnight glucose patterns. The average next-morning glucose concentrations remained within the normal physiological range (~85–95 mg/dL) across all treatments (*p* = 0.58; Figure 2).

**Figure 1.**
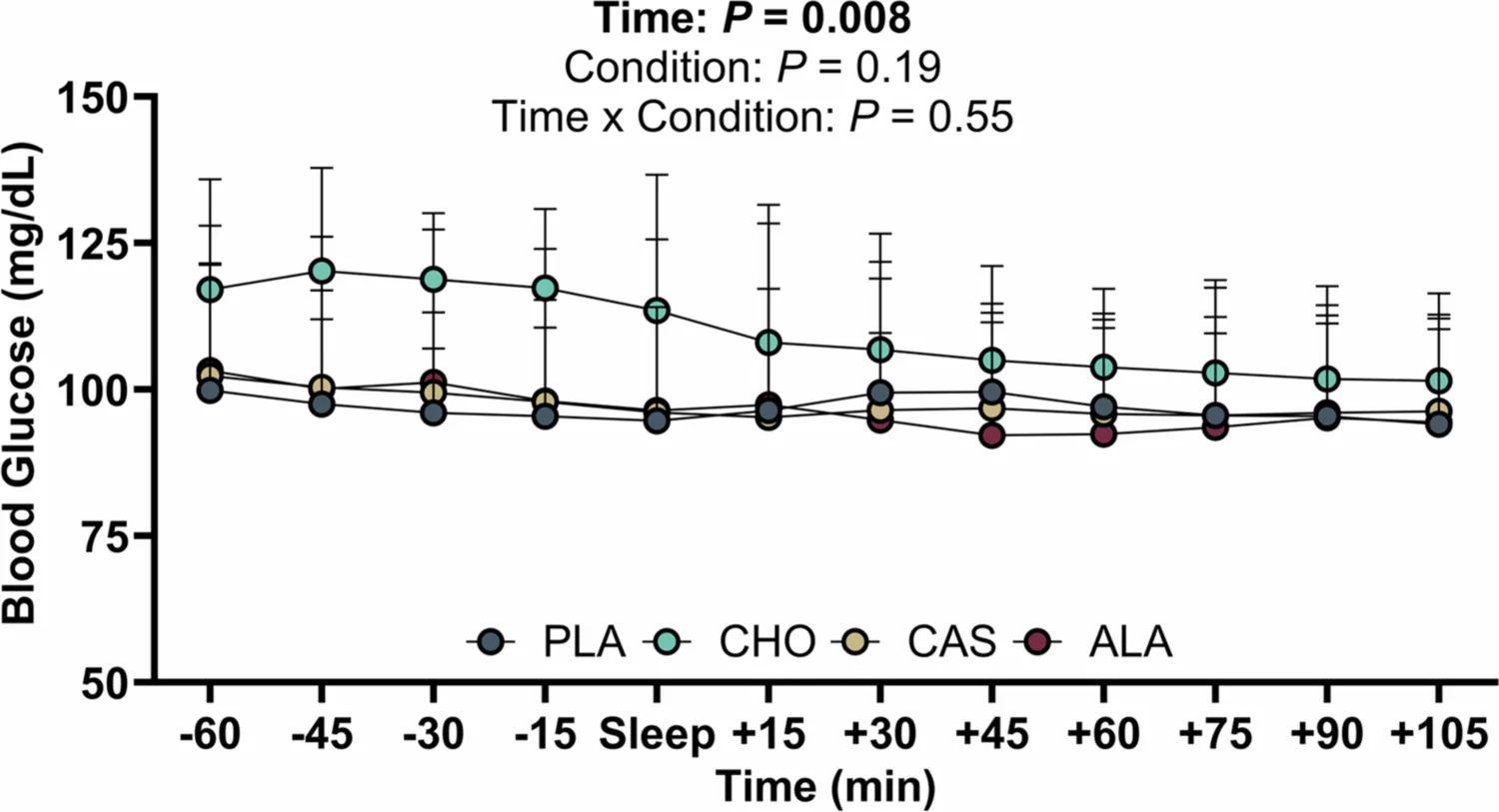
Nocturnal blood glucose dynamics, **for the CGM subsample (*n* = 6)**, across all 4 treatments from 60 minutes before and 105 minutes post objective sleep time via WHOOP 4.0 device. There was a significant effect of time for nocturnal blood glucose**, but no effect of condition.**

**Figure 2.**
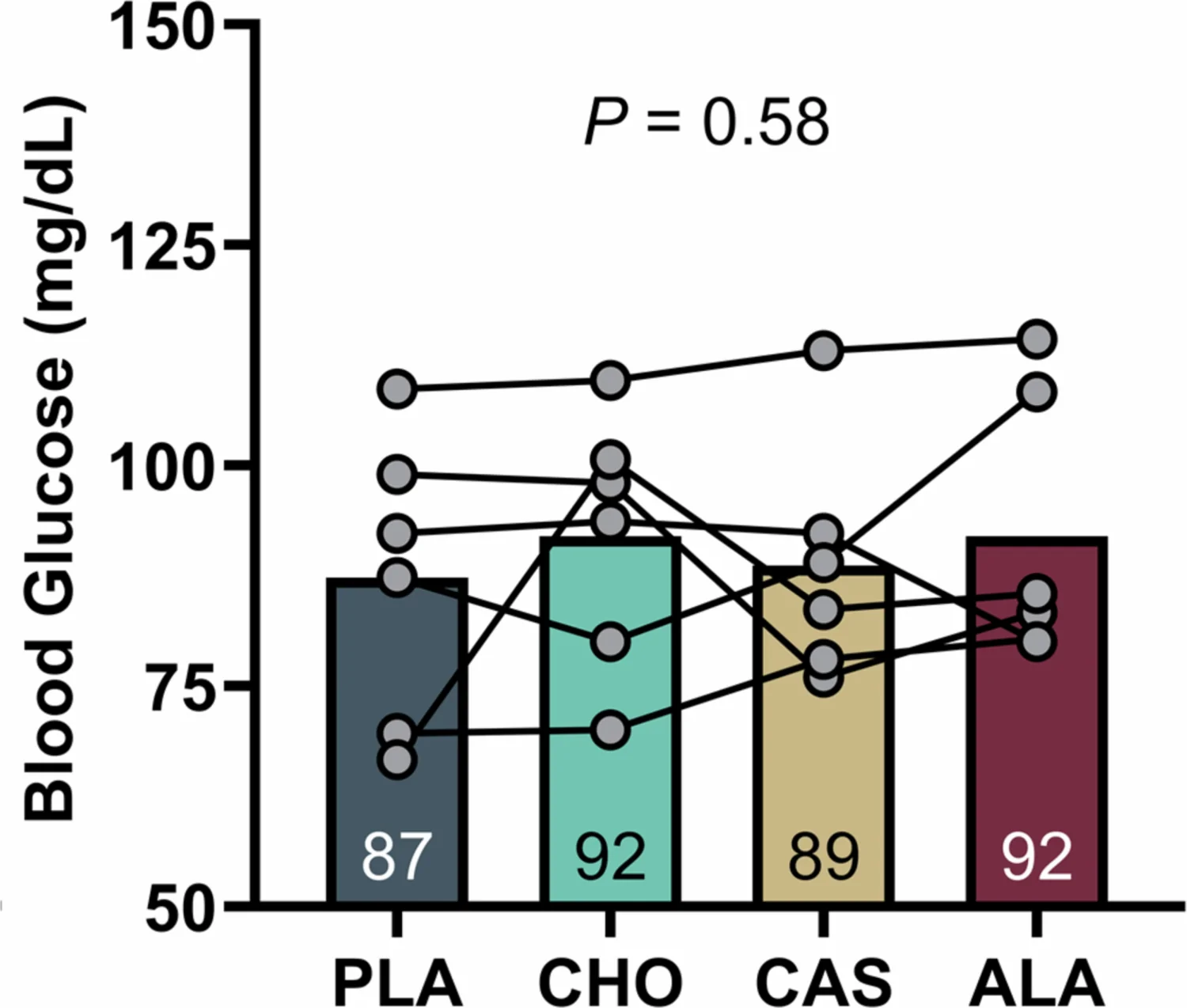
Average next morning blood glucose values **for the CGM subsample (*n* = 6).** There was no difference in morning blood glucose between conditions at reported wake time.

### 3.3. Dietary intake analysis

Participants who completed ≥75% (9/12) of the assigned food logs were included in the dietary intake analysis. Seventeen participants met this criterion, yielding 195 analyzable daily logs. Reported values reflect habitual daily intake and exclude study-provided supplements. Accordingly, participants in the CAS and ALA treatments consumed an additional 40 g of protein (160 kcal), those in the CHO treatment consumed an additional 40 g of carbohydrate (160 kcal), and the PLA contained no protein or carbohydrate and contributed 5 kcal from minor ingredients (non-nutritive sweeteners, organic acids, and natural colorants). Average daily energy intake (excluding supplements) was 2,040 ± 463 kcal. Mean macronutrient intakes were 107 ± 28 g protein (1.7 ± 0.5 g·kg^−1^), 215 ± 49 g carbohydrate (3.2 ± 0.8 g·kg^−1^), and 85 ± 21 g fat (1.3 ± 0.3 g·kg^−1^). Subjects were accurate at 35.71% when asked which treatment they believed they were consuming.

## 4. Discussion

The primary finding of this study was that three consecutive nights of pre-sleep ingestion of CAS, ALA, or CHO did not impair objective or subjective sleep or recovery measures compared with PLA in NCAA Division I female athletes. These results indicate that pre-sleep feeding (PSF) of 40 g of ALA, CAS, or CHO does not alter objective sleep quantity in elite female athletes. Similarly, subjective outcomes, such as perceived sleep quality, sleep onset latency, and next-morning recovery, remained unchanged. Taken together, this suggests that in these elite female athletes, eating a small bolus of CHO or protein (160 Kcals) before bed, regardless of macronutrient type, did not affect their sleep or how they felt upon waking.

Our findings align with previous reports indicating that protein ingestion before sleep does not impact sleep quality or quantity. In 2018, Trommelen and colleagues reported no differences in sleep time, sleep onset latency, sleep duration, sleep efficiency, or wake bouts following pre-sleep ingestion of a 30 g placebo, protein, or protein + 2 g of free Leucine in 36 healthy, recreationally active young men [35]. More recently, Greenwalt et al. (2023) reported that higher pre-sleep energy and protein intake across a competitive season was not associated with decrements in sleep quality or recovery in NCAA Division I female soccer players, reinforcing the practical applicability of PSF in team-sport settings [22]. Collectively, these data help counter the common notion that eating before bed is disruptive to sleep, in both quality and quantity, and performance; rather, they suggest that context is critical, including nutrient type, dose, and population.

The present study adds to the growing, yet mixed, body of evidence on *α*-lactalbumin (ALA) and sleep. ALA, standardized to approximately 43% of the protein formulation in the current intervention, contains a higher tryptophan fraction than other dairy proteins and has been proposed to enhance serotonin and melatonin synthesis, thereby improving sleep onset and quality [21]. Two studies in elite female rugby players reported modest but significant benefits with a ~93% ALA isolate [24,25]. Miles et al. observed improved sleep efficiency (+2.9%), reduced sleep-onset latency (−7.4 min), and enhanced next-day reaction time following evening ALA ingestion compared with casein [25]. Gratwicke et al. similarly found increases in total sleep time (+18 min) and subjective sleep quality after three weeks of nightly ALA supplementation during competition [24]. In contrast, more recent trials using lower ALA concentrations or different dosing strategies have not demonstrated measurable effects. Barnard et al. reported no differences in total sleep time, sleep efficiency, or next-morning performance between ALA, casein, or placebo in trained athletes with sleep complaints, while other field data in team-sport athletes show comparable null results when ALA is consumed at lower relative purity [26]. Together, these findings suggest that both ALA concentration and baseline sleep status may determine responsiveness, with higher-purity (∼93%) formulations showing modest benefits in sleep-compromised athletes, whereas the 43% ALA used in the present study did not affect sleep or recovery, as determined by SE and HRV; likely due to the well-established rest and recovery strategies of Division I female athletes.

Carbohydrate ingestion before sleep has previously been associated with shortened sleep-onset latency, particularly when consumed approximately four hours before bedtime [7,8], though findings in athletes are inconsistent [9,11]. In the current study, SOL did not differ between CHO and PLA, suggesting that 40 g of pre-sleep carbohydrate intake neither improved nor impaired sleep in already well-trained, habitually monitored athletes. Thus, while carbohydrate may improve sleep onset latency in certain populations or conditions, our data do not support a consistent ergogenic or sleep-promoting role in elite female athletes.

A major strength of this study is its randomized, double-blinded, crossover design, which allowed each participant to serve as her own control, thereby reducing interindividual variability. The use of validated wearable technology provided continuous, non-invasive assessment of sleep and recovery in a naturalistic environment, reflecting real-world athlete behaviors. Moreover, including both objective and subjective outcomes provides a more comprehensive view of how PSF may influence sleep. However, several limitations should be acknowledged. Importantly, the adhesion of the CMG devices was affected by various sports activities in a majority of participants, leaving us with a sub-sample of six participants for CMG data analysis, thereby limiting the strength of conclusions drawn regarding nocturnal glucose responses. The intervention was short in duration (three nights per treatment), limiting conclusions about chronic adaptation. Participants were healthy athletes without reported sleep disorders, which may limit generalizability to athletes with poor sleep quality or clinical populations. In addition, these athletes are on elite collegiate teams and have performance staff that may already have in place ideal sleep protocols. Of note, athletes reported an average daily energy intake (excluding supplements) of 2,040 ± 463 kcal. This is likely underreporting, a lack of energy intake, or both. This might override other physiological outcomes measured. Finally, while wearables provide ecologically valid data [36,37], they are not as precise as polysomnography (PSG), the gold standard for sleep assessment.

Future studies should extend these findings by testing longer interventions, examining athletes with baseline sleep difficulties, and incorporating gold-standard PSG alongside validated wearables. Additional work is also needed to explore sex-specific responses to PSF, given hormonal influences on sleep architecture and autonomic regulation across the menstrual cycle [31]. In addition, there is no data indicating a specific caloric load at which pre-sleep feeding shifts from beneficial to detrimental. Nor do we know much about the quality of foods eaten before bed (rather than the quantity).

## 5. Conclusion

From a practical perspective, the absence of negative effects of pre-sleep nutrition on sleep is meaningful. Athletes frequently struggle to meet daily energy and protein requirements, particularly during heavy training or competition periods. PSF provides a strategic window to deliver additional nutrients without fear of disrupting sleep, aligning with established principles of nutrient timing in athletes [38]. In the present study, none of the treatments (CAS, ALA, CHO, or PLA) differed in either objective or subjective outcomes, indicating that pre-sleep ingestion of any macronutrient type - protein, carbohydrate, or placebo - did not impair sleep or recovery. This finding reinforces the practical takeaway that late-evening nutrient intake can be safely included within an athlete’s routine. Although ALA and CAS did not confer unique advantages over CHO or PLA in our cohort, previous work has shown that pre-sleep protein ingestion can support overnight MPS, enhance muscle recovery, and contribute to performance adaptations when practiced consistently [13–15,34]. It is also possible that participants’ habitual protein intake of 1.7 g·kg^−1^·day^−1^ reduced the likelihood of observing additional benefits from the 40 g pre-sleep protein dose. However, any potential impact on sleep quality would still have been detected if present. Collectively, these observations suggest that while PSF may not acutely enhance sleep outcomes, protein-based PSF strategies remain as viable options for athletes seeking to optimize daily caloric and protein intake.
